# Not built for families: Associations between neighborhood disinvestment and reduced parental cognitive stimulation

**DOI:** 10.3389/fpsyg.2022.933245

**Published:** 2022-10-13

**Authors:** Caitlin F. Canfield, Lauren O’Connell, Richard C. Sadler, Juliana Gutierrez, Shanna Williams, Alan L. Mendelsohn

**Affiliations:** ^1^Division of Developmental-Behavioral Pediatrics, Department of Pediatrics, NYU Grossman School of Medicine, New York, NY, United States; ^2^Division of Public Health, Michigan State University College of Human Medicine, Flint, MI, United States; ^3^Michigan State University College of Human Medicine, Michigan State University-Hurley Children’s Hospital Pediatric Public Health Initiative, Flint, MI, United States

**Keywords:** neighborhood, parenting, built environment, infancy, disinvestment

## Abstract

Infants learn and develop within an ecological context that includes family, peers, and broader built and social environments. This development relies on proximal processes—reciprocal interactions between infants and the people and environments around them that help them understand their world. Most research examining predictors of proximal processes like parent-child interaction and parenting has focused on elements within the home and family. However, factors like the neighborhood built environment may also exhibit an influence, and may be particularly critical in infancy, as socioeconomic disparities in cognition and language emerge early in life. Moreover, influence from the built environment could independently exacerbate these disparities, as research indicates that neighborhood impacts may be especially relevant for families living in neighborhoods that have experienced disinvestment and therefore have been under-resourced. The current study examines these questions by determining the association of neighborhood vacancy rate and observed physical disorder—indicators of poverty, residential stability, and long-term structural discrimination—with parental cognitive stimulation among predominantly Black/African-American families in Flint, Michigan. Flint is particularly salient for this study because vacancy rates and disinvestment vary widely across the city, driven by its long-time status as a city struggling economically. Regression analyses controlling for caregiver education, mental health, and social support indicated that vacancy rate and physical disorder negatively predicted parental cognitive stimulation. Moreover, there were significant interactions between the built environment and social support, indicating that, particularly for parent-child shared reading, vacancy rate and physical disorder predicted reduced shared reading only when parents had limited social support. These results have important implications for public policy around vacant property demolition and neighborhood reinvestment programs, as they indicate that the neighborhood built environment is associated with parenting behaviors that have important impacts on infants’ learning and development.

## Introduction

Early environments are critical for children’s development (Bradley et al., 1989; Dieterich et al., 2006; Rowe, 2012; Cabrera et al., 2017). Impacts of family, neighborhood, and structural/institutional factors are evident even in early infancy (Burchinal et al., 2008), and have lasting impacts on learning, academic achievement, economic attainment, and mental and physical health (Duncan et al., 2010). Further, the impacts of the environment may be greater for children living in poverty or who otherwise face increased risk (McCartney and Berry, 2009; Ungar and Hadfield, 2019). However, while several studies have shown that neighborhood poverty levels and social factors impact parenting and child outcomes (Klebanov et al., 1994; Olofson, 2017; Li et al., 2022), few have examined the impacts of the neighborhood built environment on families with infants and young children, and even fewer examine them using precise (i.e., individual level data rather than data aggregated by zip code or census tract) and novel measures of the built environment. This study sought to fill this gap by examining the impact of built environment disinvestment at the neighborhood level on proximal processes of infant learning—namely, parenting behaviors—in Flint, Michigan, integrating tenets from Bronfenbrenner’s bioecological model, differential impact theory (DIT), and the weathering hypothesis.

### How neighborhoods impact development: Theoretical frameworks

The bioecological model (Bronfenbrenner and Morris, 2007) posits that human development takes place through reciprocal interactions between that person and the people, objects, and environments around them. These proximal processes must occur regularly or over an extended period of time and enable young children to make sense of their world. Moreover, proximal processes vary across people and environments, and their content and significance changes according to the developmental outcome under study and the time at which they take place, both in terms of the life course and the historical period. In this process-person-context-time (PPCT) framework (Tudge et al., 2009), proximal processes, such as parenting behaviors, are critical for infant learning and influence infant development in different ways depending on both characteristics of the infant and their environment.

Environmental and contextual factors may further differentially impact infants and their parents. Differential impact theory (Ungar, 2017) focuses on how these factors affect epigenetic, neurological, cognitive, and socioemotional development. DIT includes three principles: that the environment can affect and change people at multiple levels (e.g., biological, psychological, social); that an individual’s outcome is dependent on their level of risk exposure; and that an understanding of the multisystemic nature of individual outcomes is necessary in order to determine how to improve wellbeing. And yet, measurement of the built environment is often limited by poorly geocoded individual data or aggregated environmental data (for example, data compiled at a ZIP code or county level rather than at an address level).

Finally, the weathering hypothesis (Forde et al., 2019) suggests that chronic exposure to social and economic disadvantage leads to suboptimal health outcomes, including earlier onset of physical health conditions. In the U.S., this weathering accounts at least in part for health disparities for Black/African-American citizens who are more likely, because of systemic racism, to have lower education and income, and to live in disadvantaged neighborhoods. However, conceptualizations of weathering are often limited to contemporary measures of socioeconomic status, rather than longer time-horizon considerations of historical structural racism in the built and social environment. Further, while this has primarily been applied to physical health outcomes, there is evidence that such chronic exposure impacts proximal processes for infant cognitive development (Luke et al., 2009), as the disadvantage experienced by Black/African-American and other marginalized parents makes it more difficult for them to engage in responsive parenting behaviors like shared reading and teaching.

Taken together, these theories indicate that the community context plays a critical role in human development, both through exposure to risk and disadvantage, and through impacts on proximal processes that underlie physical, cognitive, and socioemotional growth. When considering infant learning, therefore, we would expect neighborhoods and other environments to have both direct impacts on infants’ development, and indirect effects through impacts on parenting behaviors, such as play and shared reading, that promote cognitive development. Novel methodological advances in the measurement of the neighborhood environment will allow for a more nuanced understanding of this association.

### Neighborhoods and development in infancy and early childhood

Research examining the impacts of neighborhoods on children and families has primarily focused on broad measures of neighborhood advantage or disadvantage. These studies provide preliminary support for the theoretical framework above. For instance, early research on the effects of neighborhood poverty on maternal characteristics and behaviors indicated that the proportion of neighborhood residents with low incomes was significantly associated with the physical environment within the home and warmth between the mother and child (Klebanov et al., 1994). Similarly, effects of poverty at the neighborhood level, including increased social disorder and lower levels of social cohesion, have been associated with increased parenting stress (Franco et al., 2010). On the other hand, neighborhood affluence is associated with more positive parenting practices (e.g., reduced physical discipline) through effects of increased neighborhood resources and services (Shuey and Leventhal, 2017).

Neighborhood disadvantage also has impacts on learning and development in young children. For instance, neighborhood disorder is associated with disrupted sleep at age one and through that association with decreased language development at age five (Li et al., 2022). Further, research has found specific impacts of the neighborhood environment on brain development. For instance, Hyde et al. (2020) found that although family income, maternal education, and neighborhood poverty were all related to performance on a go/no-go inhibitory control task, only neighborhood poverty was related to neural activity during the task. They further found that the proportion of families living below poverty and the median family income of a neighborhood uniquely predicted amygdala reactivity to ambiguity. Children living in neighborhoods marked by these indicators of poverty showed a heightened amygdala response to neutral faces, indicating increased emotional reactivity. Finally, infants born prematurely and living in high-risk neighborhoods were more likely than their peers in low-risk neighborhoods to have neurodevelopmental impairment, or a cognitive or language delay at age two, even after adjusting for maternal education, non-English-speaking parent, gestational age, presence of medical complications associated with prematurity, and breastfeeding (Nwanne et al., 2022).

### The specific impacts of the built environment

Much of the prior research in this field has looked broadly at poverty or at social risks within neighborhoods. However, other neighborhood attributes, such as institutional resources (e.g., libraries, child care facilities and schools, healthcare facilities, services and retailers), have important impacts on families and children’s development (Wei et al., 2021). The neighborhood built environment may be an additional factor that influences learning and development. Several studies have examined the impact of the built environment on children’s physical health. These have indicated that more walkable neighborhoods, including those with safe sidewalks, crossings, and destinations that are reachable by foot, bicycle, or public transportation, are associated with increased physical activity (Villanueva et al., 2016), and that air pollution and other environmental exposures closely related to the built environment are associated with health outcomes, including fetal growth and child respiratory health (Gascon et al., 2016).

There is reason to believe that influences of the built environment go beyond physical health alone, and may disrupt the proximal processes critical to early development in the same way that broader neighborhood indicators do. For instance, living in areas with fewer main, high-speed roads has been associated with a decreased risk for developmental vulnerability (Christian et al., 2017), and increased access to nature and green spaces has been linked to improved child mental health (Alderton et al., 2019). Further, the increased physical activity associated with walkable neighborhoods may also benefit young children’s cognitive development, and such neighborhoods support social resources, such as social networks and collective efficacy, which promote responsive parenting and children’s socioemotional wellbeing (Villanueva et al., 2016). On the other hand, neighborhoods that have experienced disinvestment and economic decline often lack the community resources, such as green space, clean air, safe streets, and even grocery stores, that have previously been shown to have positive impacts on children’s development (Walker, 2021). These same places are associated with lower rates of collective efficacy, social capital, psychological wellbeing, stability, and other elements important to the healthy development of families (Walker, 2021; Berg et al., 2022). This lack of physical and social infrastructure translates to higher rates of neighborhood stressors, and increased exposure to psychosocial risks, which, in turn leads to increased parent stress and mental health symptoms, reduced parental capacity to provide cognitively stimulating experiences (Cuellar et al., 2015), and more negative interactions with their children (Morrison Gutman et al., 2005). Parenting is thus more challenging in such places, particularly when viewed through the historical lens of practices like redlining that marginalized such communities even before physical and social disorder set in Berg et al. (2022).

#### Neighborhood context in Flint

In the city of Flint, most neighborhoods were built during one of two industrial boom periods during which the General Motors car company grew rapidly: the 1920s and 1950s. As such, Flint is characterized by aging, single-family homes from one of two eras, few multi-family apartment buildings, and an absence of row houses. The community was very much built around the automobile, but a parks plan from the 1920s afforded the city an abundance of greenspace even during its peak population in the 1960s. In subsequent decades, corporate and state disinvestment led to population decline, and this low density environment is now even more sparsely populated. Much informal greenspace is unmaintained or overgrown as a result of a lack of capacity in civic infrastructure. Many blocks which once housed dozens of homes have lost a third to half of their housing stock, and associated businesses, schools, and other institutions have closed as a result (Sadler et al., 2020).

Neighborhood disinvestment and disorder, and specifically high proportions of vacant properties as is common in Flint, have been shown to impact mental health and wellbeing in older children and adolescents. Vacant and unmaintained properties have been associated with higher rates of illness, as well as increased numbers of accidents and injuries (Brisbon et al., 2005; McDonell and Skosireva, 2009). Further, qualitative studies have indicated the significant impact of vacant properties on adolescents’ mental health, particularly anxiety and feelings of hopelessness (Teixeira, 2015). Previous studies in Flint have provided similar findings in adults, with vacancy rates related to mental health outcomes, particularly in the context of reduced social ties (Pearson et al., 2019). However, despite indications of the importance of the built environment for development in infancy and early childhood, previous studies have not examined the impacts of disinvestment, measured through vacancy rates and physical disorder, for this age group.

### The current study

The current study examined the influence of neighborhood built environment on proximal processes related to infant learning, including parent-child shared reading, parent verbal responsivity (PVR), and parent teaching behaviors, through a secondary analysis of a longitudinal randomized controlled trial (RCT) of an early childhood preventive parent-child intervention. The first aim of this study was to examine direct effects of the built environment on these cognitively stimulating parenting behaviors. The second aim was to determine whether these effects were moderated by parental social support, as previous research in Flint indicated that the built environment was particularly important for adults who lacked social ties (Pearson et al., 2019). This analysis extends previous research on the built environment in childhood by looking beyond physical health outcomes to determine the impact of neighborhood disinvestment on parenting behaviors related to infant learning and development.

## Materials and methods

This study was a secondary analysis of data from a longitudinal RCT examining the efficacy of the Video Interaction Project (VIP) intervention in Flint, MI with linkages to geocoded physical environment data. This study was registered with clinicaltrials.gov (NCT03945552), and IRB approval was obtained with the NYU Grossman School of Medicine IRB acting as the single IRB for the study (#s18-01347).

### Video interaction project (VIP)

VIP is an evidence-based primary preventive intervention that takes place in pediatric primary care clinics at the time of well-child visits. It aims to reduce disparities in early school readiness and child development through promotion of early relational health. Conceived as an enhancement to Reach Out and Read (Needleman et al., 1991), which provides children’s books and anticipatory guidance around shared reading during well-child visits, VIP adds a bachelor’s level coach who meets with families one-on-one and video-records the parent and child interacting with a toy or book provided by the program. The coach then reviews the video together with the parent, identifying and reinforcing responsive parenting behaviors and talking with the parent about ways to expand those behaviors. To further support self-reflection and active observation, the interventionist also engages the parent in discussion of their child’s development and provides a personalized pamphlet highlighting parent goals for interacting with their child at home. Each VIP session lasts 25–30 min, and participants can receive 14 sessions between birth and age three.

### Participants

Infants and their primary caregivers were enrolled in the RCT at the Hurley Children’s Clinic within 3 months of their first in-person pediatric visit. For most participants, this occurred within the first 3 months of life [Mean age at enrollment = 1.56 m (1.52)]. Inclusion criteria for children were: Gestational age of 32 weeks or more and a birthweight of at least 1,500 g; singleton birth; no known or suspected significant genetic abnormalities, neurodevelopmental disorders, neuromuscular conditions, or visual/hearing impairment; and no significant neonatal or medical complications. Inclusion criteria for caregivers were: English speaking; had custody of the child; did not have a significant communication impairment; and planned to continue receiving pediatric care at the Hurley Children’s Clinic. Families were randomized to receive VIP or care-as-usual.

To date, 78 families have completed baseline and 9-month assessments, of which 68 were currently living in the city of Flint and had neighborhood vacancy rate and observed physical disorder data available, comprising the analytic sample. Descriptive statistics can be found in Table 1. Families were primarily low-income and about two-thirds were Black/African-American.

**TABLE 1:** Demographic characteristics and descriptive statistics for the study sample (*n* = 68).

	Analytic sample % (n)
Female child	50% (34)
First-time parent	35% (24)
Parent high school graduate	82% (55)
Income-to-need ratio < 2^a^	87% (45)
**Parent race/ethnicity**	
Black/African-American	58% (39)
White	21% (14)
Other	1% (2)
Multiracial	19% (13)
**Marital status**	
Married	15% (10)
Living with partner	38% (26)
Non-cohabiting partner	15% (10)
Single	31% (21)
Receives public assistance	96% (65)

	**M (SD)**

**Maternal mental health**	
PROMIS depression	4.68 (5.27)
PROMIS anxiety	6.14 (5.59)
Perceived stress scale	13.42 (7.08)
Social support/resources	2.71 (0.34)

^a^*n* = 52.

### Procedure

Caregivers completed baseline assessments at the time of enrollment and randomization and a follow-up assessment when infants were 9 months of age. At both timepoints, caregivers were interviewed about their family and life circumstances, including sociodemographics, perceptions of their neighborhood, material hardship, stress and mental health, and resilience and self-efficacy. At baseline, caregivers also completed direct assessments of literacy and health literacy. At 9 months, caregivers answered additional questions about their parenting beliefs and behaviors.

### Measures

#### Neighborhood built environment

##### Vacancy rate

Flint’s residential landscape is predominantly comprised of single family homes. Vacancy rate was therefore calculated by taking the number of vacant residential parcels (i.e., parcels on which no home sits, where a demolition would have been conducted) and dividing it by the total number of residential parcels, using data from Genesee County, MI.

##### Neighborhood inventory for environmental typology

The neighborhood inventory for environmental typology (NIfETy) is a valid, reliable, in-person rater-based assessment used to characterize the quality of the built environment along multiple domains including physical features, disorder, violence, alcohol, and other drug exposures (Furr-Holden et al., 2008, 2010). Created in Baltimore, Maryland, it has now been deployed twice in the Flint area (Smart et al., 2021). Based on an assessment of NIfETys at 440 randomly selected block faces throughout the city, we conducted areal interpolation to predict NIfETy values for physical disorder at every location in the city, affording us the ability to append estimated NIfETy scores to every person in our sample living within the city of Flint.

#### Parent cognitive stimulation

Parent cognitive stimulation in the home was measured via maternal interview using the StimQ_2_ Cognitive Home Environment (StimQ_2_; Mendelsohn et al., 2011). The StimQ_2_ is a standardized interview measure of caregiver cognitive stimulation and includes scales assessing Reading Activities (READ), PVR, Parental Involvement in Developmental Advance (i.e., teaching activities; PIDA), and Availability of Learning Materials (ALM).

The StimQ_2_ has been validated for use with low-income populations. To ensure accuracy and limit social desirability bias, interview questions include prompts for descriptions/examples and follow-up questions. The StimQ_2_ has been shown to have high concurrent validity with the HOME Inventory and high internal consistency, with Cronbach’s alpha ranging from 0.88 to 0.93 (Dreyer et al., 1996; Mendelsohn et al., 2011; More information can be found at https://med.nyu.edu/pediatrics/developmental/research/belle-project/stimq-cognitive-home-environment). The StimQ_2_ provides an overall score (range 0–39), as well as scores for subscales mentioned above. For the current study, analyses were conducted for overall StimQ_2_ scores, as well as for the READ (range 0–18), PVR (range 0–16), and PIDA (range 0–5) subscales of the Infant version of the measure, in order to examine nuances in relations between built environment and parenting behavior.

#### Social support

Social support was assessed using the Social Resources subscale of the Resilience Scale for Adults. This subscale includes 7 items that ask respondents to rate which of two statements better describes them (e.g., “I can discuss personal issues with no one” vs. “I can discuss personal issues with friends/family members”). The Social Resource subscale has high internal consistency (α = 0.87) and concurrent validity with the Tromso Social Intelligence Scale (Friborg et al., 2003).

#### Covariates

##### Caregiver education

Caregiver education was included as a continuous variable reflecting years of formal schooling. Education was chosen as a covariate, rather than income, because it’s relation to cognitive stimulation has been well-established (e.g., Magnuson, 2007; Zadeh et al., 2010) and because more than 15% of participants in the current study chose not to answer questions related to income. Analyses including income-to-need ratio are included in Supplementary material.

##### Caregiver mental health

An indicator for caregiver mental health was created using confirmatory factor analysis based on scores from three validated scales at infant age 9 months: PROMIS depression, PROMIS anxiety, and Perceived Stress Scale (PSS). Both the PROMIS depression and anxiety scales consist of 8 items that ask the respondent to indicate how often they experienced mood-related symptoms (e.g., “I felt worthless,” “I felt uneasy”) in the past 7 days. Both scales have strong reliability and high correlations with other validated mental health measures (Cella et al., 2010). The PSS contains 10 items that ask about the frequency with which respondents feel their lives are unpredictable, they are overloaded, or things are beyond their control. A systematic review indicated that the PSS has high internal consistency (α = 0.60–0.91) and adequate external consistency (*r* = 0.23–0.70; Lee, 2012).

##### Randomization group

Group assignment was included as a binary variable, scored 0 (*control*) or 1 (*VIP*).

### Analysis plan

Bivariate analyses and multivariate OLS regression analyses were used to examine whether the neighborhood built environment, measured via vacancy rate and NIfETy physical disorder observations, predicted parenting behaviors. Separate regressions were conducted for each measure of built environment. The false discovery rate was used to control for multiple comparisons across measures, and thus *q*-values, rather than *p*-values, are presented for individual predictors. All records were geocoded to their precise home location using ESRI’s World Geocoding Service, allowing us to spatially join all related built environment data at the exact location where the child lived. While we acknowledge the importance of spatial polygamy in shaping a diversity of exposures (Matthews and Yang, 2013), we also lean on the fact that most exposure takes place close to the home, particularly for young children. In order to examine spatial patterns, geocoded StimQ_2_ scores were mapped along with smoothed residential vacancy rate areas using ArcGIS. In spatial representations, locations of geocoded addresses were randomly adjusted, or “jittered,” and enlarged to preserve confidentiality. Finally, based on previous findings for adults in Flint (Pearson et al., 2019), we also examined whether there was an interaction between the neighborhood built environment and social support in predicting parenting behaviors. Significant interactions were further analyzed using the Johnson-Neyman procedure to identify regions of significance of the conditional effect of vacancy rate and observed physical disorder on parenting behaviors.

## Results

### Descriptive statistics

Table 1 provides the demographic characteristics and descriptive statistics for the study sample. Approximately one-third of participants were first-time parents, and the majority were from low-income households and identified as Black/African-American or multiracial. Overall, mothers reported relatively few mental health symptoms and moderate levels of social support. Importantly, there were no differences in these characteristics between the analytic sample and the full sample.

As shown in Table 2, caregivers engaged in a moderate to high number of cognitively stimulating parenting behaviors, including moderate levels of reading and verbally responsive activities, and high rates of teaching activities. Vacancy rates in residential neighborhoods in Flint are relatively high as compared to other American cities, with a median rate in the current sample of approximately 8%. Rates vary widely across census tracts, however, ranging from nearly 0% in many neighborhoods immediately east and west of downtown Flint to well above 80% in many neighborhoods directly north of downtown.

**TABLE 2:** Descriptive statistics and correlations between primary study variables.

	*M* (*SD*)	1.	2.	3.	4.	5.	6.
1. Vacancy rate	0.16 (0.21)	–					
2. NIfETy physical disorder	–0.03 (0.58)	0.71**	–				
3. StimQ_2_ total score	21.47 (6.42)	−0.38**	−0.30*	–			
4. StimQ_2_ read	8.89 (4.23)	−0.33**	−0.28*	0.86**	–		
5. StimQ_2_ PVR	9.28 (2.72)	−0.24*	–0.21	0.73**	0.33**	–	
6. StimQ_2_ PIDA	3.14 (1.26)	–0.15	–0.10	0.60**	0.29**	0.53**	–

**p < 0.01; *p < 0.05.

Bivariate correlations indicated that both vacancy rate and observed physical disorder were significantly associated with StimQ_2_ total scores, as well as scores on the StimQ_2_ read subscale. Vacancy rate was also associated with PVR, measured by the StimQ_2_ PVR subscale.

### Built environment and parenting

**FIGURE 1:**
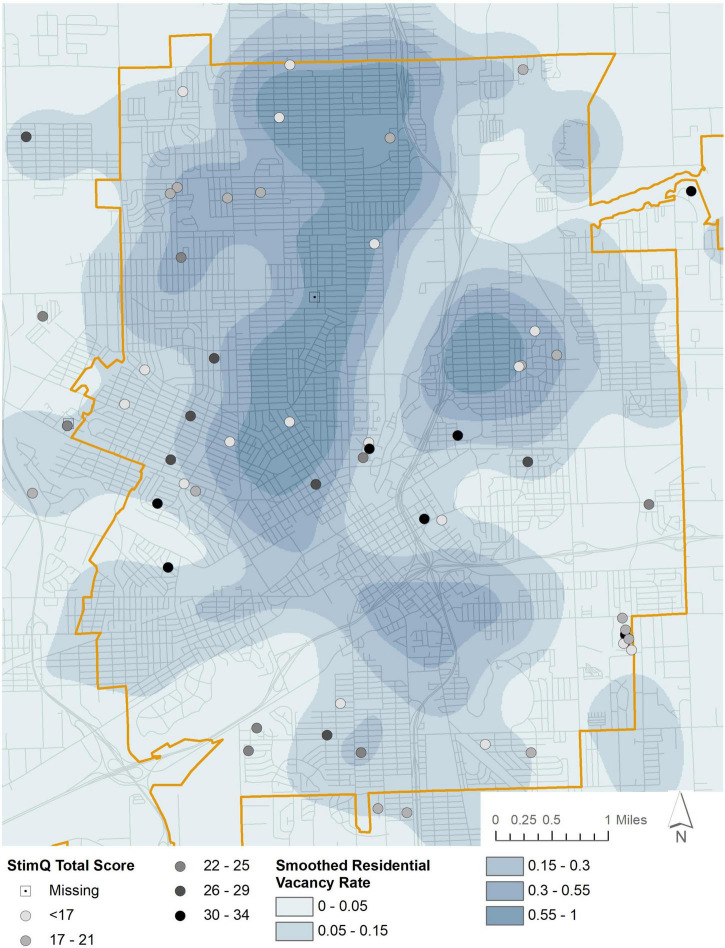
Parental cognitive stimulation (StimQ_2_) and neighborhood vacancy rates.

We examined effects of vacancy rate using a series of OLS regression models predicting parent StimQ_2_ scores and controlling for randomization group, and caregiver mental health, social support, and education (Table 3). For total StimQ_2_ scores, the model including vacancy rate was significant, [*F*(5, 57) = 6.00, *p* < 0.001], and explained 29% of the variance in StimQ_2_ scores. Vacancy rate, maternal education, and maternal social support were all significant independent predictors of mothers’ cognitively stimulating behavior. Figure 1 demonstrates how vacancy rate itself can be seen to vary spatially, with lower rates east, west, and southwest of downtown, and higher rates to the north and northeast. Further, average StimQ_2_ scores showed similar variation, ranging from 23.62 (4.97) when vacancy rates were less than 5% to 13.2 (4.92) when vacancy rates were above 55% (Figure 1).

**TABLE 3:** Standardized regression coefficients for models predicting parent cognitively stimulating behaviors from neighborhood vacancy rates.

	Total stimq_2_	StimQ_2_ read	StimQ_2_ PVR	StimQ_2_ PIDA
Vacancy rate	−0.29*	−0.23^†^	−0.22^†^	–0.06
Maternal education	0.42**	0.34**	0.26*	0.32**
Maternal mental health	–0.13	–0.06	–0.17	−0.24*
Social support	−0.24*	–0.08	−0.30*	–0.16

**q < 0.01; *q < 0.05; ^†^*q* < 0.10.

In examining StimQ_2_ subscales, vacancy rate approached significance in predicting reading and verbally responsive activities (*q* = 0.096 for both), but was not a predictor of parent teaching behaviors.

**FIGURE 2:**
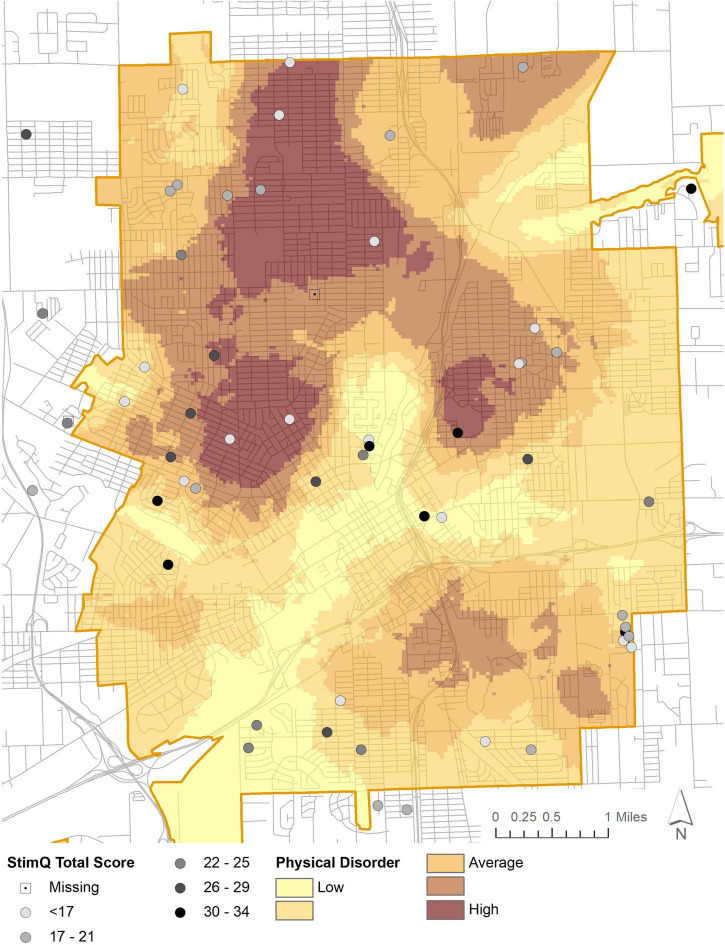
Parental cognitive stimulation (StimQ_2_) and NIfETy physical disorder.

The model predicting StimQ_2_ Total scores from NIfETy physical disorder scores and other sociodemographic predictors was also significant and predicted 23% of the variance in StimQ_2_ scores, [*F*(5, 36) = 3.44, *p* < 0.05]. Although NIfETy scores were a marginally significant predictor of parenting behavior before adjusting for multiple comparisons, it was no longer a significant predictor after adjusting (Table 4). However, both physical disorder and StimQ_2_ scores vary spatially across Flint. Disorder tends to be highest north of downtown, with the lowest scores running through downtown and to the east and southwest, as indicated in Figure 2. Average StimQ_2_ scores varied across levels of physical disorder, from 21.85 (6.36) when disorder was high, to 24.20 (6.81) when disorder was low. NIfETy scores were not a significant predictor of any of the StimQ_2_ subscales.

**TABLE 4:** Standardized regression coefficients for models predicting parent cognitively stimulating behaviors from observed physical disorder.

	Total stimq_2_	StimQ_2_ read	StimQ_2_ PVR	StimQ_2_ PIDA
NIfETy physical disorder	–0.28	–0.26	–0.20	–0.06
Maternal education	0.42**	0.37*	0.28	0.35*
Maternal mental health	–0.17	–0.10	–0.17	–0.17
Social support	–0.25	–0.09	−0.32*	–0.20

**q < 0.01; *q < 0.05.

### Interactions between built environment and social support

**FIGURE 3:**
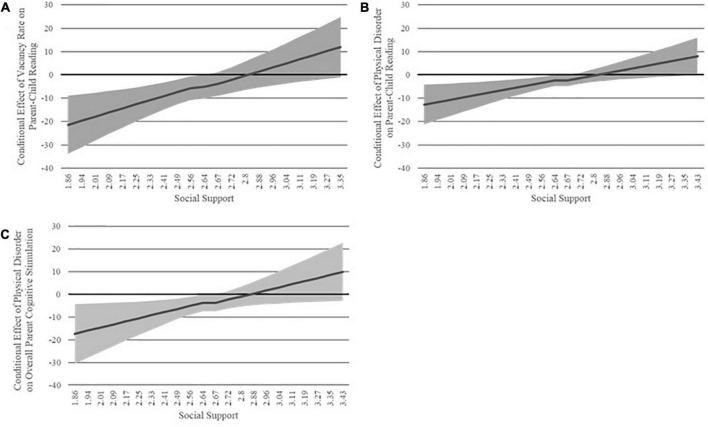
Johnson-Neyman plots indicating regions of significance for the conditional effect of **(A)** vacancy rate on parent-child reading, **(B)** observed physical disorder on parent-child reading, and **(C)** observed physical disorder on overall parent cognitive stimulation across levels of social support.

To examine the second aim of this study, OLS regressions with a multiplicative interaction term were used to examine whether the effects of vacancy rate and observed physical disorder on parenting were conditional on parents’ levels of social support. For brevity, only significant interactions are discussed here. Results for all analyses can be found in Supplementary material. The interaction between vacancy rate and social support was significant in predicting scores on the StimQ_2_ Read subscale, β = 2.65, *p* < 0.01. Johnson-Neyman procedure indicated that the conditional effect of vacancy rate on parent-child reading was significant only when social support was low (Figure 3). The interaction between observed physical disorder and social support was significant in predicting both total StimQ_2_ scores, β = 3.70, *p* < 0.05, and StimQ_2_ Read scores, β = 4.49, *p* < 0.05. As above, the Johnson-Neyman procedure indicated that the conditional effect of physical disorder on parent-child reading and on parents’ cognitively stimulating behavior overall was significant only when social support was low (Figure 3).

## Discussion

This study demonstrates the importance of the neighborhood built environment for infant’s learning and development. Specifically, these findings suggest that vacancy rates and physical disorder in an infant’s immediate neighborhood are associated with fewer parental cognitively stimulating behaviors, proximal processes that are critical for infants to learn about and understand the world around them. Moreover, these measures of neighborhood disinvestment were particularly salient in the context of low social support, as interactions indicated that they were only predictive when social support scores were low. This mirrors findings of the impact of the built environment on adult mental health in Flint, and underscores the impacts that the neighborhood built environment has on neighborhood residents, both directly and indirectly.

The present results are consistent with prior work demonstrating the impact of neighborhood disadvantage on children’s physical, cognitive, and socioemotional development, and on research examining the impacts of the built environment on adult and adolescent physical and mental health. This study extends this understanding by demonstrating that the built environment has associations not just with traditional health outcomes, but for parenting behaviors that are closely tied to infant learning and development. Further, these associations were independent of families’ income-to-need (see Supplementary material). The current findings also extend understanding of the importance of well-designed built environment measures. We used individual-level vacancy rates and objectively measured, individual-level physical disorder scores to determine associations between parenting behaviors and the built environment. This is in contrast to previous work that has mainly relied on subjective, aggregated, or simplified measures of the built environment, such as zip code level data or overall wealth derived from census data. We also report here the approximate (jittered) home locations of participants, both for conceptual understanding and as a proposal as to the possibilities of obtaining and mapping residential data for participants in medical research. Highlighting approximate home locations (rather than aggregating up to ZIP codes or some other unit) also allows researchers to see specific parts of neighborhoods where many people have participated in the program, thus enabling opportunities for geographic recruitment in underserved and well-served neighborhoods alike.

Although the current study was not powered to examine direct impacts of vacancy rates on infant learning, preliminary analyses of a subsample of infants (*n* = 28) who completed the Mullen Scales of Early Learning at 9 months of age indicated that vacancy rates were associated with lower scores in expressive language (β = –0.49, *p* < 0.05); this suggests that the neighborhood built environment may impact infants’ learning as well as the proximal processes that make that learning possible, and is a promising avenue for future research.

These findings closely connect with our team’s prior and continuing work evaluating structural racism in the built environment and how legacies of disinvestment can negatively impact health and wellbeing many decades after the initial land use decision was made to segregate, disinvest, or otherwise create disadvantage in a neighborhood (Rabinowitz et al., 2020; Sadler et al., 2021). The present findings have important implications for clinicians, others working with families in communities that have been under-resourced, and health and urban policymakers alike. Understanding the impact that neighborhoods have on parents and parenting behavior can inform programs and encourage providers to take the full complexity of children’s experience at the individual, family, and community level into account. These findings also indicate the importance of policies to address disinvestment at the neighborhood, city, and state levels. Past authors have remarked on the social justice dimensions of dealing with high vacancy neighborhoods in ways that do not further disadvantage the residents therein (e.g., Németh and Langhorst, 2014; Sadler et al., 2020). Historical approaches included urban renewal, which forcibly moved hundreds of thousands of people out of (typically inner-city) neighborhoods, an act carried out in the name of neighborhood improvement or redevelopment (Hyra, 2012). In the contemporary sense, cities are faced with overstrained/underutilized infrastructure, while dealing with the repercussions for residents living in such spaces for decades. Intergenerational epigenetic effects of living in such weathered neighborhoods have been established in studies of physical health (Forde et al., 2019), and the current analyses provide a first indication that such effects may be important for infant learning as well.

In one series of studies in Flint, authors explored the potential negative implications of conducting some version of right-sizing (or infrastructure removal/down-sizing) on high vacancy neighborhoods (Audirac and Hackworth, 2021; Dewar, 2021; Ehrenfeucht and Nelson, 2021; Ryan, 2021; Sadler et al., 2020). These studies indicated that policies such as those that have been implemented historically achieve neither efficiency or equity for residents, and that community-based approaches are needed. Clearly, the present work has important connections not only to the field of child development and our understanding of proximal processes for infant learning, but also to the growing evidence base on the importance of person-centered approaches to planning for population decline in places such as Flint.

This study is not without limitations. Because of the COVID-19 pandemic, in-person assessments in this study were necessarily limited during the years 2020–2022, leading to a more limited sample size. Further, the city of Flint has experienced both chronic and acute trauma through ongoing disinvestments and the Flint Water Crisis. Given this unique experience, findings may not generalize to other cities or populations. Future research will continue to examine the impact of neighborhood and city disinvestment on cognitive, physical, and socioemotional development among infants and children in Flint, as well as whether disrupted proximal processes, such as the cognitively stimulating parenting behaviors examined here, may, in fact, be modifiable factors, with programs promoting responsive parent-child interactions helping to buffer the effects of a chronic lack of resources in families’ communities.

The present findings provide an important first step in developing a comprehensive model of the effects of families’ neighborhoods on infant and child development. While much previous research has focused on general socioeconomic factors, such as overall neighborhood wealth, this is the first analysis to our knowledge that uses precise built environment data to examine how vacancy rates and physical disorder—signs of chronic disinvestment—impacts proximal processes critical to infant learning and cognitive development. Continued development of this model is essential to ensuring optimal development during childhood and continued wellbeing throughout the lifespan.

## Data availability statement

The datasets presented in this article are not readily available as the data collection is ongoing. Datasets will be available upon completion of primary study aims by request to the corresponding author. Further inquiries should be directed to the corresponding author.

## Ethics statement

The studies involving human participants were reviewed and approved by the NYU Grossman School of Medicine IRB. Written informed consent to participate in this study was provided by the participants and/or the participants’ legal guardian/next of kin.

## Author contributions

CC led the writing of the manuscript, with significant contributions from LO’C and RS. AM gave valuable feedback and suggestions. JG and SW provided support during the data collection and writing of the manuscript. All authors contributed to the article and approved the submitted version.
