# Supplementary material for: Not built for families: Associations between neighborhood disinvestment and reduced parental cognitive stimulation
Source: Front Psychol. 2022 Oct 13;13:933245. doi: 10.3389/fpsyg.2022.933245 (PMC9606826; doi:10.3389/fpsyg.2022.933245)
Supplement: Supplementary file 1 [file Table_1.PDF]

Supplementary Material

1 Additional Analyses including Income-to-Need as a Covariate.

Supplementary Table 1. Bivariate correlations between income-to-need ratio and other variables of interest.

n = 66	*r*
Maternal Education	.52**
Vacant	-.22†
NIfETy Physical Disorder	-.27†
StimQ Total Score	.32**
StimQ Read	.28*
StimQ PVR	.26*
StimQ PIDA	.13

** $p < .01$; * $p < .05$; † $p < .10$

Supplementary Table 2. Standardized regression coefficients for models predicting parent cognitive stimulation from vacancy rates, including income-to-need ratio.

	Total $StimQ_2$	$StimQ_2$ Read	$StimQ_2$ PVR	$StimQ_2$ PIDA
Vacancy Rate	-0.32*	-0.25†	-0.18	-0.06
Income-to-Need	0.27*	0.21	0.24†	0.13
Maternal Mental Health	-0.21	-0.13	-0.22	-0.25†
Social Support	-0.24	-0.06	-0.25†	-0.04

* $p < .05$; † $p < .08$

Supplementary Table 3. Standardized regression coefficients for models predicting parent cognitive stimulation from physical disorder, including income-to-need ratio.

	Total $StimQ_2$	$StimQ_2$ Read	$StimQ_2$ PVR	$StimQ_2$ PIDA
NIfETy Physical Disorder	-0.21	-0.22	-0.05	-0.14
Income-to-Need	0.24	0.17	0.23	0.14
Maternal Mental Health	-0.30	-0.24	-0.22	-0.26
Social Support	-0.05	0.04	-0.14	-0.01

* $p < .05$; † $p < .08$

2 Additional Analyses Examining Interactions between Neighborhood Disinvestment and Social Support

Supplementary Table 4. Standardized regression coefficients for models including the interaction between vacancy rate and social support.

	Total $StimQ_2$	$StimQ_2$ Read	$StimQ_2$ PVR	$StimQ_2$ PIDA
Vacancy Rate	-1.56†	-2.87**	0.21	0.86
Maternal Education	0.42**	0.36**	0.26*	0.32*
Maternal Mental Health	-0.11	-0.02	-0.17	-0.26*
Social Support	-0.38*	-0.36*	-0.25	-0.06
Vacancy * Social Support	1.27	**2.65****	-0.43	-0.92

** $p < .01$; * $p < .05$; † $p < .08$

Supplementary Table 5. Standardized regression coefficients for models including the interaction between NIfETy physical disorder and social support.

	Total $StimQ_2$	$StimQ_2$ Read	$StimQ_2$ PVR	$StimQ_2$ PIDA
NIfETy Physical Disorder	-3.95*	-4.72**	-2.19	-0.78
Maternal Education	0.38*	0.32*	0.26	0.34*
Maternal Mental Health	-0.06	0.03	-0.11	-0.15
Social Support	-0.16	-0.004	-0.28†	-0.19
NIfETy * Social Support	**3.70***	**4.49***	2.01	0.73

** $p < .01$; * $p < .05$; † $p < .08$
